# From porphyrin benzylphosphoramidate conjugates to the catalytic hydrogenation of 5,10,15,20-tetrakis(pentafluorophenyl)porphyrin

**DOI:** 10.3762/bjoc.10.54

**Published:** 2014-03-10

**Authors:** Marcos C de Souza, Leandro F Pedrosa, Géssica S Cazagrande, Vitor F Ferreira, Maria G P M S Neves, José A S Cavaleiro

**Affiliations:** 1 Departamento de Química Orgânica, Universidade Federal Fluminense, 24020-141 Niterói, RJ, Brasil,; 2 Department of Chemistry & QOPNA, University of Aveiro, 3810-193 Aveiro, Portugal

**Keywords:** catalytic hydrogenation, chlorin, isobacteriochlorin, phosphoramidate, porphyrin

## Abstract

Three new porphyrin aminoalkyl dibenzylphosphoramidates were synthesized by nucleophilic aromatic substitution of one *p*-fluorine atom of 5,10,15,20-tetrakis(pentafluorophenyl)porphyrin (**TPPF_20_**) by primary aminoalkyl dibenzylphosphoramidates. The nucleophilic aromatic substitution was promoted by microwave irradiation in *N*-methyl-2-pyrrolidinone. Attempts to remove the benzyl groups of the phosphoramidate moiety by hydrogenolysis with 10% Pd/C led to the cleavage of the P–N bond and the reduction of the macrocycle to hydroporphyrin-type derivatives. The extent of the effect of the catalytic hydrogenation to **TPPF_20_** with 10% Pd/C was then studied with a variety of solvents. The results showed that ethanol/DMF is the solvent of choice to produce chlorin **TPCF_20_** and an ethanol/DMF/NEt_3_ mixture is more adequate to produce isobacteriochlorin (**TPIF_20_**).

## Introduction

**Scheme 1:**
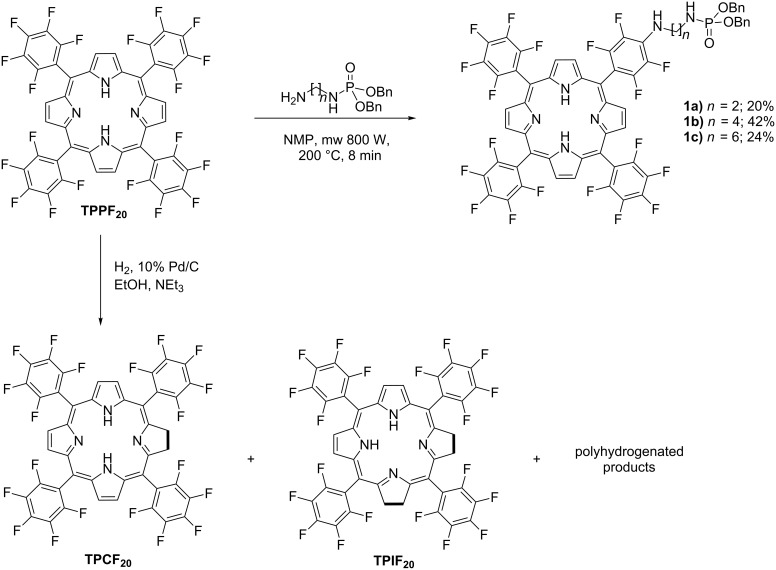
Synthesis of porphyrin aminoalkylphosphoramidates **1a–c**, and of chlorin (**TPCF_20_**) and isobacteriochlorin (**TPIF_20_**) from **TPPF_20_**.

The use of porphyrin derivatives as photosensitizers is considered for the photodynamic therapy (PDT) of malignant tumors and the treatment of age-related macular degeneration in several countries [1–2]. It is already known that the efficiency of the drug greatly depends on the porphyrin amphiphilicity, which must provide good interactions with the lipid membranes and the physiological medium. Consequently, tumor localization and better selectivity can be achieved by introducing polar substituents [3]. For this reason, we recently synthesized porphyrin diisopropylphosphoramidate conjugates derived from 5,10,15,20-tetrakis(pentafluorophenyl)porphyrin (**TPPF_20_**). Such compounds showed a high photostability and a good capacity to generate singlet oxygen, but their solubility in water is moderate [4]. Additionally, we tried to convert the diisopropyl phosphoramidate ester moiety into the corresponding phosphoramidic acid in order to improve the hydrophilicity. Conventional hydrolytic methods, such as heating under reflux in 6 N HCl or the use of ClTMS/NEt_3_, were unsuccessful, as the P–N bond instead of the P–O bond was cleaved [5–6]. This undesired result pointed to the synthesis of a phosphoramidate derivative different from the diisopropyl one. Here, we describe the synthesis of the analogue porphyrin dibenzylphosphoramidates **1a–c**. Attempts to remove the benzyl groups by mild hydrogenolysis over 10% Pd/C led to the reduction of the macrocycle, thereby affording chlorin and other derivatives (Scheme 1). Considering the importance of **TPPF_20_** as a template for further functionalizations [7] and the better suitability of chlorins for PDT compared to porphyrins due to their enhanced red-shifted Q bands [2], we decided to investigate the direct hydrogenation of **TPPF_20_** with H_2_ in the presence of 10% Pd/C.

## Results and Discussion

### Synthesis of porphyrin dibenzylphosphoramidates **1**

The method [4] for the nucleophilic aromatic substitution of the *p*-fluorine atoms of **TPPF_20_** under reflux in toluene/triethylamine did not work in acceptable yields when aminoalkyl dibenzylphosphoramidates were used as nucleophiles instead of the diisopropylphosphoramidate analogues [7–8]. Prolonged times with heating under reflux (20–46 h) needed to promote the substitution caused the decomposition of the dibenzylphosphoramidates to a great extent, rendering the method inadequate. Thus, we performed the reaction by microwave irradiation witth a solution of **TPPF_20_** and the primary aminoalkyl dibenzylphosphoramidates in *N*-methyl-2-pyrrolidinone (NMP) as the solvent [9]. The conversion into the new porphyrin monoaminoalkyldibenzylphosphoramidate conjugates **1a–c** was achieved in eight minutes in 20%, 42% and 24% yields, respectively. Longer reaction times led to the formation of polysubstituted products. A significant advantage for the reaction under microwave irradiation is its applicability even in the presence of functional groups, which are prone to decompose under prolonged heating times.

For all derivatives **1a–c** the parent ions [M + H]^+^ can be seen in ESI–MS spectra. The NMR spectra confirm the substitution of one *p*-fluorine atom. The eight β-pyrrolic protons in ^1^H NMR are split into two doublets with two protons each and one singlet with four protons in the range of 8.8–9.0 ppm. The inner NH protons appear as broad singlets at approximately −2.9 ppm. The ^19^F NMR spectra showed a differentiation between the eight *o*-fluorine atoms, leading to two signals with six F and two F at approximately −159.0 ppm and −164.0 ppm, and between the eight *m*-fluorine atoms, leading to another set of signals with six F and two F at approximately −185.0 ppm and −183.0 ppm. The three remaining *p*-fluorine atoms gave one signal at −175.0 ppm. ^31^P NMR signals appeared at approximately 10 ppm, corresponding to the phosphoramidate group. The three compounds **1a–c** showed very similar UV–vis spectra, indicating that the substitution of the electron withdrawing *p*-fluorine atom with the electron-donating amine phosphoramidate did not cause significant changes (see Supporting Information File 1).

**Figure 1:**
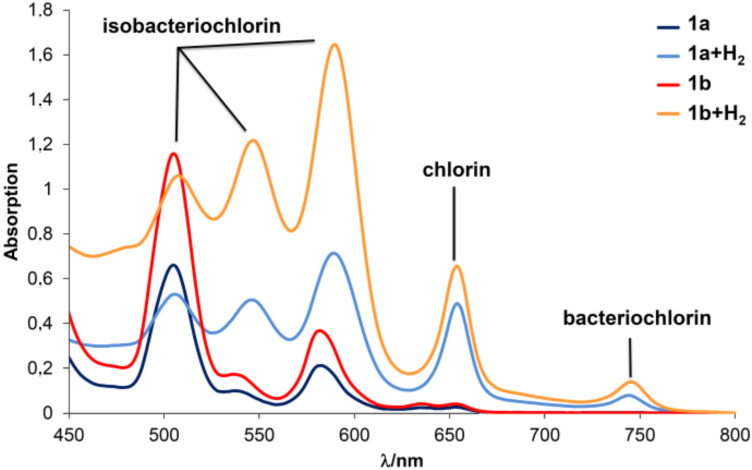
UV–vis spectrum (CHCl_3_) of the crude mixture obtained from the hydrogenation of **1a** and **1b** over 10% Pd/C and triethylamine.

Attempts to selectively remove the benzyl groups of the porphyrin dibenzylphosphoramidates **1a** and **1b** by hydrogenolysis were carried out with H_2_ and 10% Pd/C in the presence of triethylamine in order to establish a mild environment for the catalyst [10]. However, when the porphyrin dibenzylphosphoramidates were subjected to these conditions the spectroscopic analysis (UV–vis, MS, ^31^P NMR and ^1^H NMR) of the crude mixture allowed to consider the loss of the dibenzylphosphoryl group [–P(O)(OBn)_2_] and the reduction of the macrocycle core. This chemical transformation on the macrocycle core was easily detected from the electronic spectrum of the crude mixtures (Figure 1), which shows one Q band at 654 nm, one set of three Q bands from 500–600 nm, and another smaller one at 750 nm. These Q bands are characteristic of chlorin, isobacteriochlorin and bacteriochlorin derivatives, respectively [11]. We concluded that under the conditions of catalytic hydrogenolysis with 10% Pd/C and NEt_3_ the benzyl groups could not be selectively removed without affecting the phosphoramidate moiety and the porphyrin core.

### Catalytic hydrogenation of TPPF_20_ with H_2_/10% Pd/C

There are many methods for performing the reduction of porphyrins (or metalloporphyrins) leading to different levels of hydrogenation on the β-pyrrolic double bonds [12–13]. Most of these methods employ chemicals other than H_2_ as hydrogen donors to reduce porphyrins into chlorins [14–18]. In our case, we decided to investigate the extent of the direct catalytic hydrogenation with H_2_ over **TPPF_20_** in the presence of 10% Pd/C and triethylamine before proceeding with the hydrogenolysis of the porphyrin dibenzylphosphoramidates **1**. Moreover, there are no reports concerning the reduction of this porphyrin with H_2_/10% Pd/C to produce the corresponding chlorin or isobacteriochlorin.

The experiments were carried out at room temperature, and the reaction mixture was protected from light. Hydrogen was bubbled directly into the stirring suspension of 20 mg of porphyrin and 10 mg of catalyst with different solvents, such as ethanol, THF, CHCl_3_ and CH_2_Cl_2_, and alternating the presence of triethylamine in each case. The use of ultrasound was necessary to improve the solubility of the porphyrin when ethanol was the solvent.

**Figure 2:**
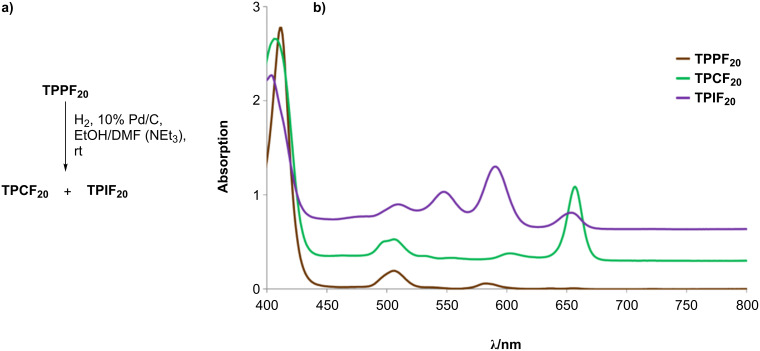
Main products from the hydrogenation of **TPPF_20_** with 10% Pd/C. Comparative UV–vis spectra of isolated **TPPF_20_**, **TPCF_20_** and **TPIF_20_** in CHCl_3_.

The progress of the reaction was monitored by UV–vis and TLC in petroleum ether/CHCl_3_ (4:1). When finished the catalyst was filtered off, and the solvent was evaporated. In THF, CHCl_3_ and CH_2_Cl_2_ no modification was observed in the TLC of the reaction mixture after 48 hours, and the porphyrin was completely recovered. The addition of NEt_3_ did not change these results. In the experiments in ethanol it was observed that the presence of NEt_3_ improved the solubility of the porphyrin and the color of the solution changed from brown to violet after a few minutes of hydrogenation. Besides the brown spot on the TLC plate related to non-converted **TPPF_20_**, a green and a violet prominent spot appeared, which were attributed to the corresponding chlorin (**TPCF_20_**) and isobacteriochlorin (**TPIF_20_**), respectively. Figure 2 represents a qualitative comparison of the UV–vis spectra of isolated **TPPF_20_**, **TPCF_20_** and **TPIF_20_** concerning their characteristic Q bands.

The total reaction mixture from the experiments in ethanol was analyzed by ^1^H NMR. The ratio **TPPF_20_**:**TPCF_20_**:**TPIF_20_** was then calculated by correlating the integrals of selected aromatic β-pyrrolic protons of the three macrocycles [19] in the region between 7 and 9 ppm (Table 1). Entries 1 and 2 show that, in spite of the amount of nonreacted porphyrin after 48 hours, its conversion into **TPCF_20_** is considerable. Complementary experiments were carried out in ethanol with 2% DMF as the solvent with the purpose of refining the conditions to generate the maximum amount of chlorin and isobacteriochlorin (Table 1, entries 3 and 4). TLC control of the reactions at 30 minutes intervals showed that the green and the violet spots immediately appeared and the amounts of the corresponding compounds were increased in the reaction mixture up to a reaction time of 16 hours. Thereafter, a number of polar colored components could be seen on the TLC plate in detriment of **TPCF_20_** and **TPIF_20_**. It must be noted that the amount of unreacted **TPPF_20_** was markedly smaller than in previous experiments. At this point we inferred that the conversion of **TPCF_20_** into **TPIF_20_** benefits from the presence of NEt_3_ in the solution as can be seen from the proportion of isobacteriochlorin formed in entry 3 (Table 1). When NEt_3_ is not added to the solution of ethanol/DMF (Table 1, entry 4) the conversion of **TPPF_20_** into **TPCF_20_** is efficient, but the subsequent formation of **TPIF_20_** is not particularly pronounced. This suggests that ethanol/DMF is the solvent of choice for preferentially obtaining **TPCF_20_** and ethanol/DMF/NEt_3_ is more adequate to produce **TPIF_20_**.

**Table 1:** Experimental details for the hydrogenation of **TPPF_20_** and proportion of the unreacted **TPPF_20_**, **TPCF_20_** and **TPIF_20_** in the mixture.

Entry	Solvent	Time	NEt_3_ (Y/N)^a^	Proportion^b^ **TPPF_20_**:**TPCF_20_**:**TPIF_20_**		

1^c^	ethanol	48 h	Y	54	36	10
2	ethanol	48 h	N	66	33	trace
3	ethanol/ DMF (2%)	16 h	Y	30	07	63
4	ethanol/ DMF (2%)	16 h	N	26	58	16

^a^10% in volume. ^b^Calculated from the integrals of the β-pyrrolic H. ^c^Segment of ^1^H NMR spectrum (in CDCl_3_) for the reaction mixture of entry 1.
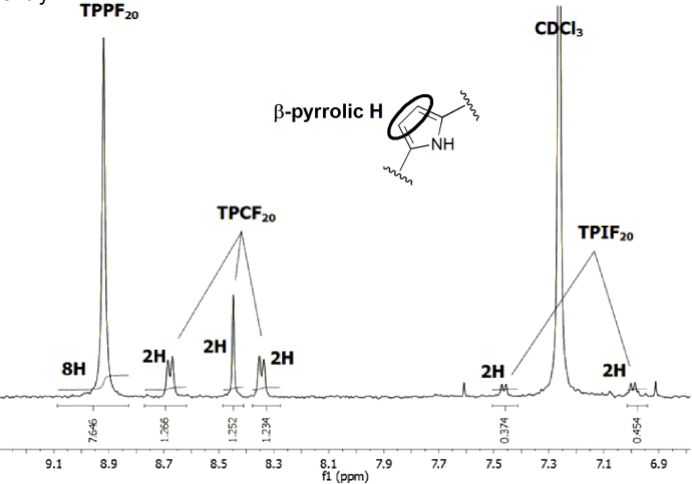

The use of NEt_3_ seems to favor the conversion of the porphyrin into isobacteriochlorin, probably by binding more efficiently the chlorin intermediate onto the catalyst surface influencing not only the stationary function of the catalyst, but also the adsorption–desorption equilibrium at that surface thus facilitating the subsequent hydrogenation step.

Purification by preparative TLC, in petroleum ether:CHCl_3_ (4:1), of each reaction mixture allowed the recovering of the unreacted porphyrin starting material. Full characterization by spectroscopic methods (^1^H NMR, ^19^F NMR, HRMS and UV–vis) was made for the fractions corresponding to **TPCF_20_** and **TPIF_20_**. The mass spectra (ESI) for both compounds showed a signal at *m*/*z* corresponding to the [M + H]^+^ ion, which is in accordance with the calculated values. The green fraction (**TPCF_20_**) showed the typical electronic spectrum of chlorins [11,20–21], with a relatively intense Q-band at 656 nm. The violet fraction (**TPIF_20_**) presented a set of three Q bands of growing intensity at 509 nm, 547 nm and 589 nm, characteristic of isobacteriochlorins (Figure 2) [11].

The chemical shifts in the ^1^H NMR spectra for the two NH protons are displaced from −2.92 ppm in **TPPF_20_** to −1.53 ppm in **TPCF_20_** and to 4.82 ppm in **TPIF_20_**. The presence of one signal due to 4H at 4.31 ppm in the chlorin case and one signal due to 8H at 3.43 ppm in the case of isobacteriochlorin is a result of the hydrogenation of one and two β-pyrrolic double bonds of the porphyrin macrocycle [19] (Supporting Information File 1).

The low conversion rates of the porphyrin macrocycle might be attributed to the tendency of the highly hydrophobic molecule to remain adsorbed on the carbon matrix, thereby lowering the progress of the reaction [22]. This was more pronounced in solvents where **TPPF_20_** is very soluble, such as CHCl_3_, CH_2_Cl_2_ and THF, because a hydrogenation reaction did not take place. In ethanol and its combinations with NEt_3_ and DMF the dynamic adsorption–desorption between the catalyst and the substrates was established in an adequate extension. The balance between the solubility of the porphyrin and its various reduced counterparts and the adsorption on the catalytic palladium surface is of great importance for the conversion to the desired **TPCF_20_** and **TPIF_20_**.

## Conclusion

Three new porphyrin aminoalkyl dibenzylphosphoramidates **1a–c** derived from 5,10,15,20-tetrakis(pentafluorophenyl)porphyrin (**TPPF_20_**) were synthesized and characterized. Attempts to remove the benzyl groups of the phosphoramidate moiety of **1a–c** by hydrogenolysis with 10% Pd/C led to cleavage of the P–N bond and to the reduction of the macrocycle to hydroporphyrin-type derivatives.

Our method of catalytic hydrogenation at the porphyrin core of the template **TPPF_20_** to **TPCF_20_** and **TPIF_20_** is cleaner and also more straightforward than most of the methods employing chemicals other than H_2_ as hydrogen donors. So far, the results indicate that the reduction is facilitated in polar medium, such as mixtures of ethanol, DMF and triethylamine, and that it is possible to tune the ratio of chlorin and isobacteriochlorin by adjusting the solvent composition. Triethylamine probably plays the role of enhancing the polarity of the medium, which in turn affects the adsorption–desorption equilibrium over the catalyst surface.

The used porphyrin **TPPF_20_** is known as a platform leading to several derivatives of biological significance [7]. The procedure reported here is a simple method for the synthesis of its chlorin **TPCF_20_** and bacteriochlorin **TPIF_20_** derivatives. Both compounds are then available as substrates for transformations leading to compounds, which are potentially biologically active.

## Supporting Information

File 1 Experimental details, characterization data for new compounds, and copies of NMR spectra.
