# Supplementary material for: From porphyrin benzylphosphoramidate conjugates to the catalytic hydrogenation of 5,10,15,20-tetrakis(pentafluorophenyl)porphyrin
Source: Beilstein J Org Chem. 2014 Mar 10;10:628–33. doi: 10.3762/bjoc.10.54 (PMC3999763; doi:10.3762/bjoc.10.54)

Supporting Information

for

From porphyrin benzylphosphoramidate conjugates to the catalytic hydrogenation of 5,10,15,20-tetrakis(pentafluorophenyl)porphyrin

Marcos C. de Souza*[1,2], Leandro F. Pedrosa[1], Géssica S. Cazagrande[1], Vitor F. Ferreira[1], Maria G. P. M. S. Neves[2] and José A. S. Cavaleiro*[2]

Address: [1]Departamento de Química Orgânica, Universidade Federal Fluminense, 24020-141 Niterói, RJ, Brasil, and [2]Department of Chemistry & QOPNA, University of Aveiro, 3810-193 Aveiro, Portugal.

Email: Marcos C. de Souza - gqomarc@vm.uff.br; José A. S. Cavaleiro - jcavaleiro@ua.pt

*Corresponding author

Experimental details, characterization data for new compounds, and copies of NMR spectra

Table of Contents

Experimental details.. S2

References.. S4

^{1}H, ^{13}C and ^{31}P NMR spectra.. S5

Experimental details

^{1}H, ^{19}F, and ^{31}P NMR spectra were recorded on a Bruker Avance 300 spectrometer at 300.13, 282.38, and 121.50 MHz, respectively. $CDCl_3$ was used as the solvent. Chemical shifts are reported in ppm (δ) and coupling constants (*J*) are given in Hz. Mass spectra and HRMS (ESI) were recorded on VG AutoSpec Q and M mass spectrometers. Absorption spectra were recorded on a Shimadzu UV-2501-PC spectrophotometer in $CHCl_3$ solutions. Flash chromatography was carried out with silica gel 35–70 mesh (Merck). Analytical TLC was carried out on precoated sheets with silica gel (0.2 mm thick, Merck). The aminoalkyl dibenzylphosphoramidates were synthesized in 67–75% yield from commercial dibenzylphosphonate and aliphatic diamines, as described by Souza et al in reference [1].

General procedure for monosubstituted $TPPF_{20}$ phosphoramidates 1a–c by microwave irradiation:

In a small vial 0.04 mmol (39.0 mg) of 5,10,15,20-tetrakispentafluorophenylporphyrin ($TPPF_{20}$) and 0.10 mmol of the aminoalkyldibenzylphosphoramidate derivatives (32.0 mg for n = 2; 34.8 mg for n = 4; 37.6 mg for n = 6) were dissolved in *N*-methyl-2-pyrrolidinone (NMP, 2.0 mL). The closed vial was irradiated in a microwave oven (Mileston, Microsynth, 800 W, 1 bar, 200 °C,) for 2 min intervals, until no starting material was visualized by TLC (usually 8 min). The mixture was diluted with chloroform, washed with aqueous $KHCO_3$ solution (3 x 5 mL) and dried over Na_2SO_4. The solvent was evaporated under reduced pressure. The crude product was purified by silica-gel column chromatography using ethyl acetate/petroleum ether 70% as the eluent.

5-(4-{[2-(Dibenzyloxyphosphorylamino)ethyl]amino}-2,3,5,6-tetrafluorophenyl)-10,15,20-tris(pentafluorophenyl)porphyrin (**1a**): Dark brown solid. Isolated yield 20%. ^{1}H-NMR δ = -2.91 (s, 2H, NH), 3.23-3.39 (m, 3H, $C\underline{H_2}N\underline{H}P$), 3.69-3.73 (m, 2H, $C\underline{H_2}NHPh$), 4.80 (br s, 1H, $CH_2N\underline{H}Ph$), 5.16 (d, *J* = *8.2*, 4H, $OC\underline{H_2}Ph$), 7.35-7.47 (m, 10H, $OCH_2\underline{Ph}$), 8.85–9.01 (m, 8H, H-β). ^{19}F-NMR δ = -185.10 to - 184.88 (m, 6F, F-*meta*), -183.86 (d, *J* = 16.9, 2F, F-*meta*), -175.10 to -174.91 (m, 3F, F-*para*), -163.95 (dd, *J* = 5.6 and 16.9, 2F, F-*ortho*), -160.00 (dd, *J* = 7.1 and 22.5, 6F, F-*ortho*), ^{31}P-NMR δ = 10.29, HRMS (ESI) *m*/*z* calcd. for

$C_{60}H_{31}F_{19}N_6O_3P$ $[M+H]^+$: 1275.1892, found: 1275.1886, UV-vis($CHCl_3$) λmax/nm (log ε) 408 (5.06), 506 (4.01), 584 (3.52).

5-(4-{[4-(Dibenzyloxyphosphorylamino)butyl]amino}-2,3,5,6-tetrafluorophenyl)-10,15,20-tris(pentafluorophenyl)porphyrin (**1b**): Dark brown solid. Isolated yield 42%. ^{1}H-NMR δ = -2.90 (s, 2H, NH), 1.63-1.79 (m, 4H, $CH_2(C\underline{H_2})_2CH_2$), 2.84-2.90 (m, 1H, N$\underline{H}$P), 2.98-3.08 (m, 2H, $C\underline{H_2}$NHP), 3.63 (t, *J* = 6.9, 2H, $C\underline{H_2}$NHPh), 5.11 (d, *J* = 7.7, 4H, $OC\underline{H_2}$Ph), 7.34-7.45 (m, 10H, $OCH_2\underline{Ph}$), 8.86–9.01 (m, 8H, H-β). ^{19}F-NMR δ = -185.10 to -184.87 (m, 6F, F-*meta*), -184.32 (d, *J* = 16.9, 2F, F-*meta*), -175.09 to -174.91 (m, 3F, F-*para*), -164.06 (dd, *J* = 5.6 and 21.2, 2F, F-*ortho*), -160.00 (dd, *J* = 5.6 and 24.0, 6F, F-*ortho*), ^{31}P-NMR δ = 10.03, HRMS (ESI) *m*/*z* calcd. for $C_{62}H_{35}F_{19}N_6O_3P$ $[M+H]^+$: 1303.2205, found: 1303.2199, UV-vis($CHCl_3$) λmax/nm (log ε) 410 (5.31), 506 (4.23), 582 (3.65).

5-(4-{[6-(Dibenzyloxyphosphorylamino)hexyl]amino}-2,3,5,6-tetrafluorophenyl)-10,15,20-tris(pentafluorophenyl)porphyrin (**1c**): Dark brown solid. Isolated yield 24%. ^{1}H-NMR δ = -2.90 (s, 2H, NH), 1.40-1.58 (m, 6H, $(C\underline{H_2})_3CH_2CH_2$NHP), 1.78 (m, 2H, $C\underline{H_2}CH_2$NHP), 2.75-2.81 (m, 1H, N$\underline{H}$P), 2.91-2.97 (m, 2H, $C\underline{H_2}$NHP), 3.65 (t, *J* = 6.9, 2H, $C\underline{H_2}$NHPh), 4.26 (br s, 1H, $CH_2N\underline{H}$Ph), 5.08 (d, *J* = 7.6, 4H, $OC\underline{H_2}$Ph), 7.33-7.40 (m, 10H, $OCH_2\underline{Ph}$), 8.87–9.03 (m, 8H, H-β). ^{19}F-NMR δ = -185.10 to -184.91 (m, 6F, F-*meta*), -184.43 (d, *J* = 14.1, 2F, F-*meta*), -175.11 to -174.96 (m, 3F, F-*para*), -164.06 (dd, *J* = 5.6 and 19.7, 2F, F-*ortho*), -160.00 (dd, *J* = 5.6 and 24.0, 6F, F-*ortho*), ^{31}P-NMR: 10.14, HRMS (ESI) *m*/*z* calcd. for $C_{64}H_{39}F_{19}N_6O_3P$ $[M+H]^+$: 1331.2518, found: 1331.2512, UV-vis($CHCl_3$) λmax/nm (log ε) 415 (5.28), 508 (4.26), 584 (3.88).

General procedure for hydrogenation of $TPPF_{20}$:

20 mg of $TPPF_{20}$ and 10 mg of Pd/C (10%) were suspended in 7 mL of the appropriate solvent and 0.7 mL of NEt_3, when was the case. Hydrogen was bubbled directly into the stirred suspension at room temperature (20–25 °C) and protected from light. At the end of the reaction the catalyst was filtered off and washed with chloroform, followed by evaporation of the solvents at a low temperature (<60 °C). The crude product was fractionated by preparative TLC on silica-gel plates using a mixture of petroleum ether/chloroform 4:1 as the eluent.

TPCF$_{20}$: ^{1}H-NMR δ = -1.53 (s, 2H, NH), 4.31 (s, 4H, CH_2), 8.34 (d, *J* = 5.0, 2H, H-β), 8.45 (s, 2H, H-β), 8.67 (d, *J* = 5.0, 2H, H-β), ^{19}F-NMR δ = -185.05 and -184.15 (2dt, *J* = 19.7 and 5.6, 8F, F-*meta*), -175.58 and -175.35 (2t, *J* = 19.7, 4F, F-*para*), -161,41 and -160.47 (2dd, *J* = 8.4 and 22.5, 8F, F-*ortho*), HRMS (ESI) *m*/*z* calcd. for $C_{44}H_{13}F_{20}N_4$ $[M+H]^+$: 977.0821, found: 977.0812, UV-vis($CHCl_3$) λmax/nm (log ε) 406 (5.09), 505 (4.01), 656 (4.59).

TPIF$_{20}$: ^{1}H-NMR δ = 3.43 (s, 8H, CH_2), 4.82 (br s, 2H, NH), 6.99 (d, *J* = 4.5, 2H, H-β), 7.46 (d, *J* = 4.5, 2H, H-β), ^{19}F-NMR δ = -185.17 to -185.03 (m, 2F, F-*meta*), -184.59 to -184.41 (m, 4F, F-*meta*), -183.45 to -183.29 (m, 2F, F-*meta*), -175.66 (t, *J* = 19.8, 2F, F-*para*), -176.18 (t, *J* = 19.8, 1F, F-*para*), -175.75 (t, *J* = 19.8, 1F, F-*para*), -162.41 (dd, *J* = 8.5 and 25.4, 2F, F-*ortho*), -162.04 (dd, *J* = 8.5 and 22.6, 4F, F-*ortho*), -161.66 (dd, *J* = 5.6 and 22.6, 2F, F-*ortho*), HRMS (ESI) *m*/*z* calcd. for $C_{44}H_{15}F_{20}N_4$ $[M+H]^+$: 979.0977, found: 979.0967, UV-vis($CHCl_3$) λmax/nm (log ε) 403 (4.56), 509 (3.70), 547 (3.84), 589 (4.07).

References

1. Souza, M. C.; Macedo, W. P.; Torres, T. S.; Pedrosa, L. F.; Alt, H. G. *Phosphorus, Sulfur Silicon Relat. Elem.* **2006,** *181,* 1885–1893. doi:10.1080/10426500500543776

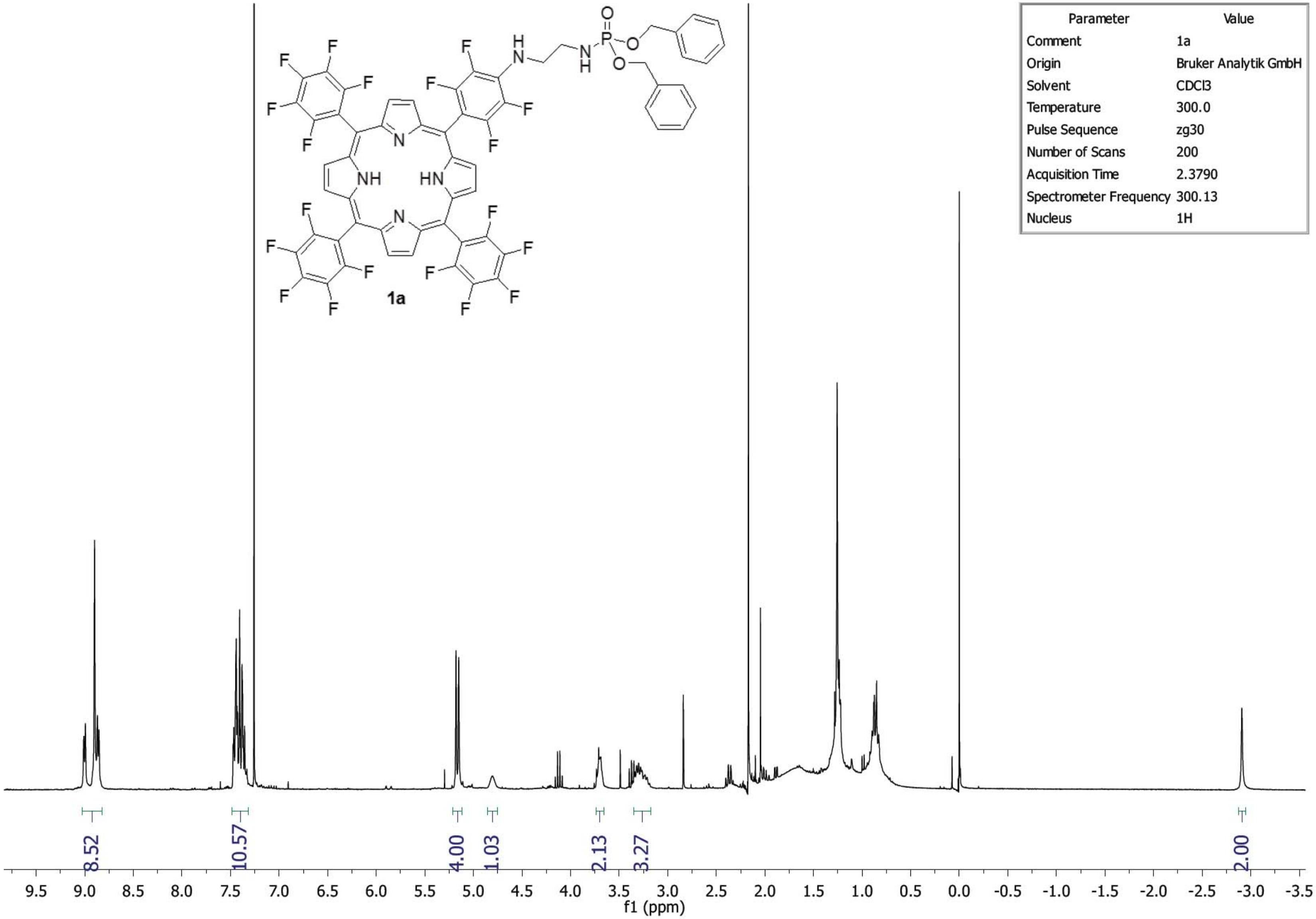
1a
Parameter Value
Comment 1a
Origin Bruker Analytik GmbH
Solvent CDCl3
Temperature 300.0
Pulse Sequence zg30
Number of Scans 200
Acquisition Time 2.3790
Spectrometer Frequency 300.13
Nucleus 1H
8.52
10.57
4.00
1.03
2.13
3.27
2.00
9.5 9.0 8.5 8.0 7.5 7.0 6.5 6.0 5.5 5.0 4.5 4.0 3.5 3.0 2.5 2.0 1.5 1.0 0.5 0.0 -0.5 -1.0 -1.5 -2.0 -2.5 -3.0 -3.5
f1 (ppm)

Parameter	Value
Comment	1a
Origin	Bruker Analytik GmbH
Solvent	CDCl3
Temperature	300.0
Pulse Sequence	zg
Number of Scans	200
Acquisition Time	0.2901
Spectrometer Frequency	282.41
Nucleus	19F

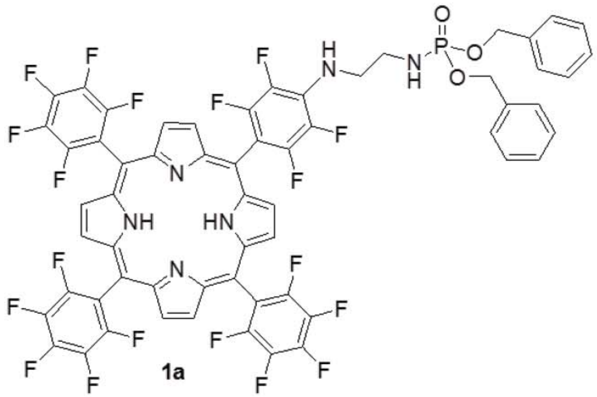

1a

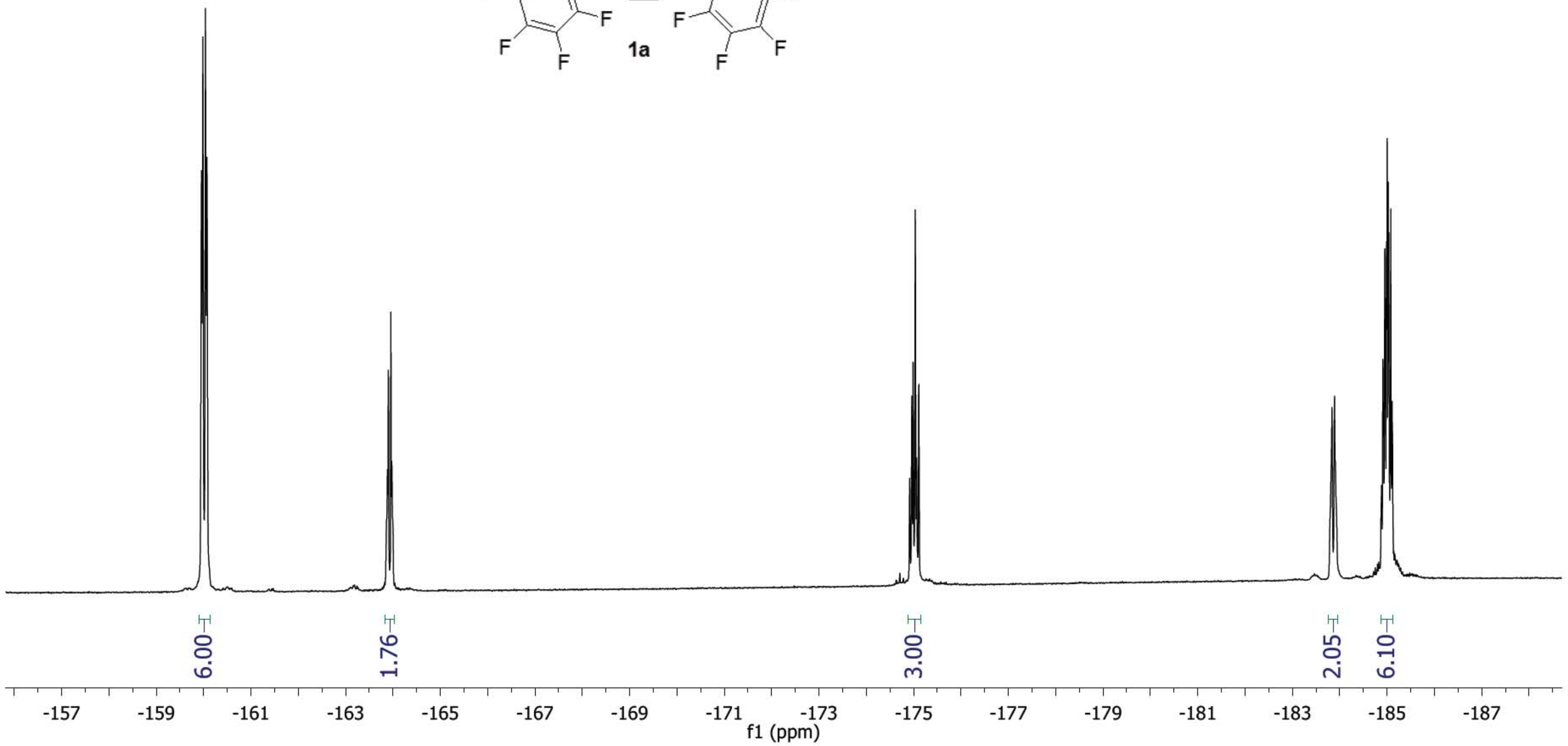

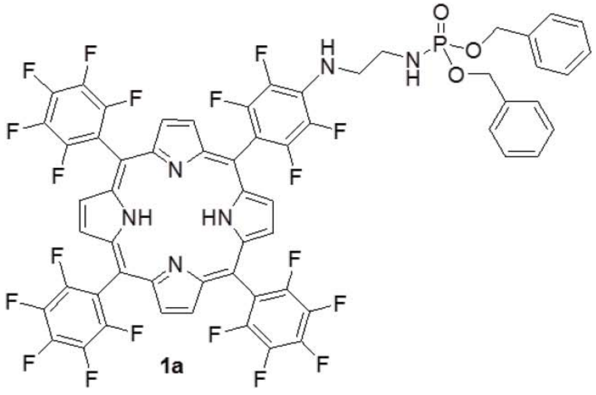

Parameter	Value
Comment	1a
Origin	Bruker Analytik GmbH
Solvent	CDCl3
Temperature	300.0
Pulse Sequence	zg
Number of Scans	200
Acquisition Time	0.6751
Spectrometer Frequency	121.49
Nucleus	31P

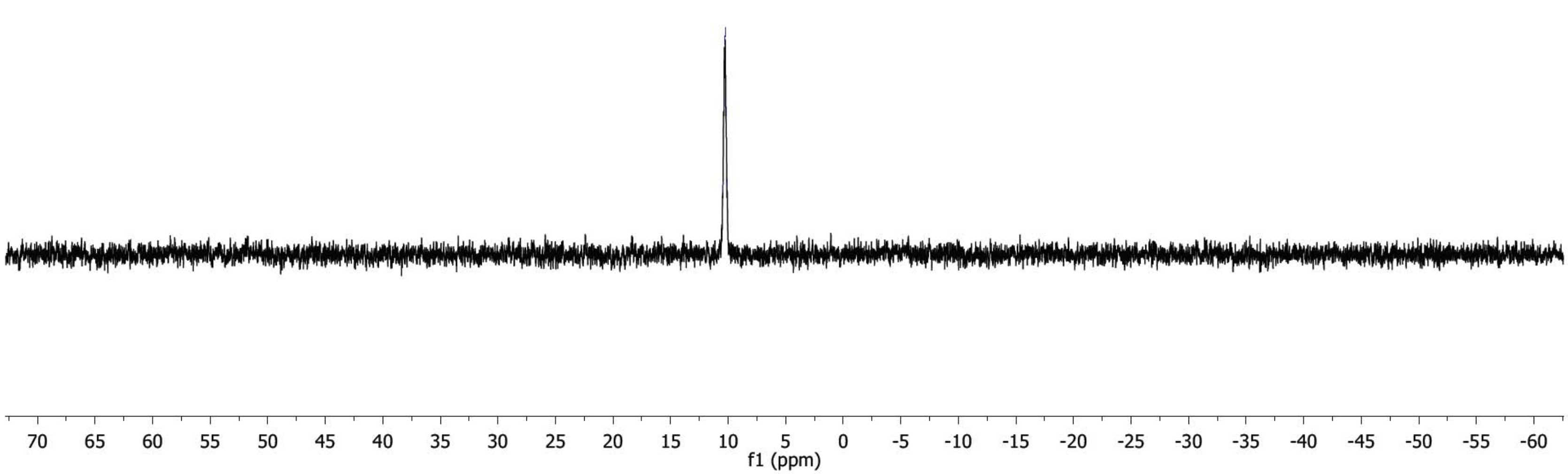

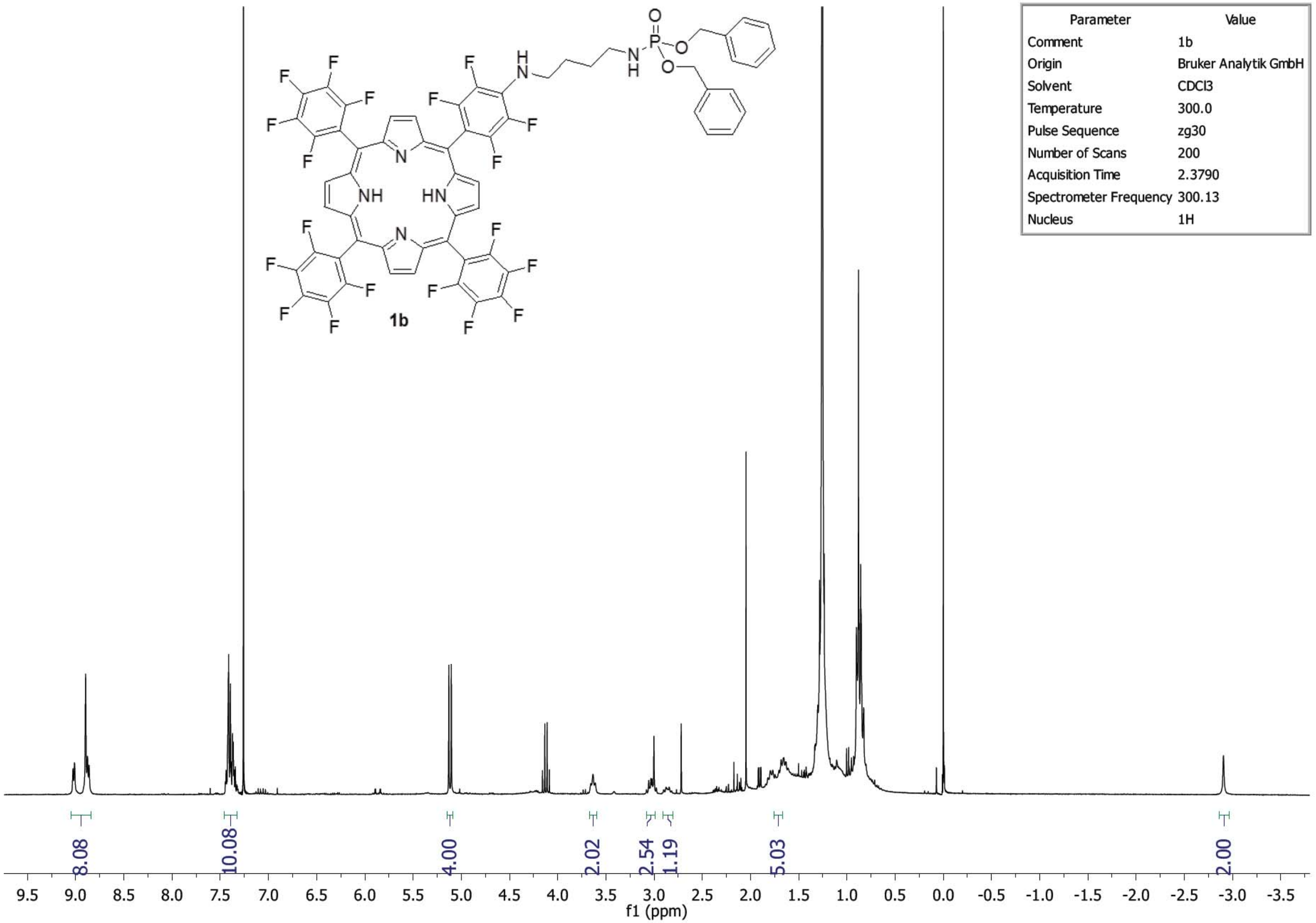

Parameter	Value
Comment	1b
Origin	Bruker Analytik GmbH
Solvent	CDCl3
Temperature	300.0
Pulse Sequence	zg30
Number of Scans	200
Acquisition Time	2.3790
Spectrometer Frequency	300.13
Nucleus	1H

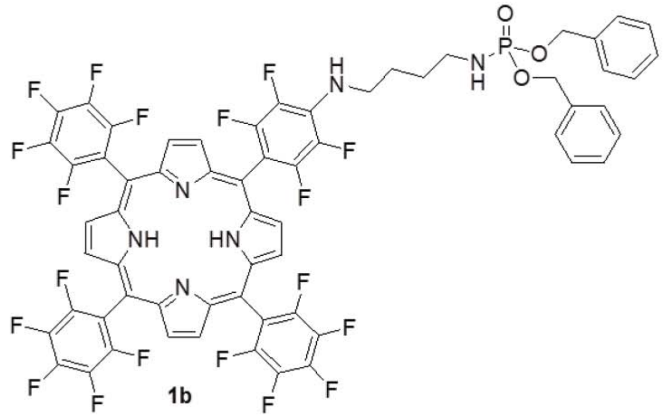

1b

Parameter	Value
Comment	1b
Origin	Bruker Analytik GmbH
Solvent	CDCl3
Temperature	300.0
Pulse Sequence	zg
Number of Scans	200
Acquisition Time	0.2901
Spectrometer Frequency	282.41
Nucleus	19F

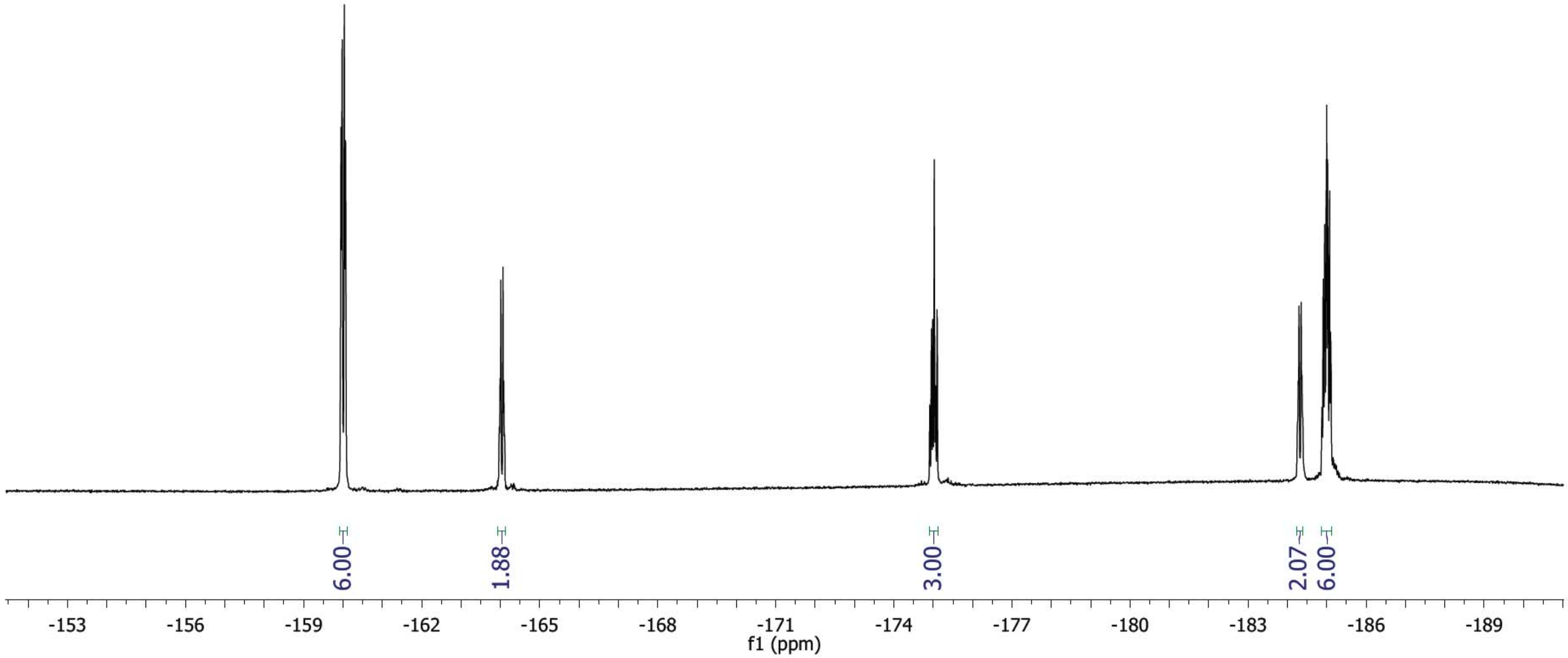

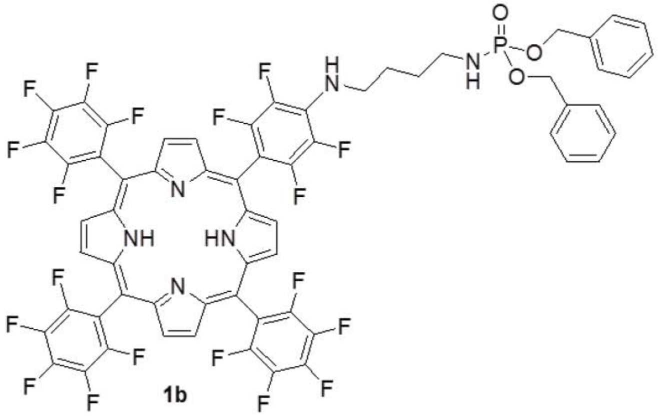

1b

Parameter	Value
Comment	1b
Origin	Bruker Analytik GmbH
Solvent	CDCl3
Temperature	300.0
Pulse Sequence	zg
Number of Scans	200
Acquisition Time	0.6751
Spectrometer Frequency	121.49
Nucleus	31P

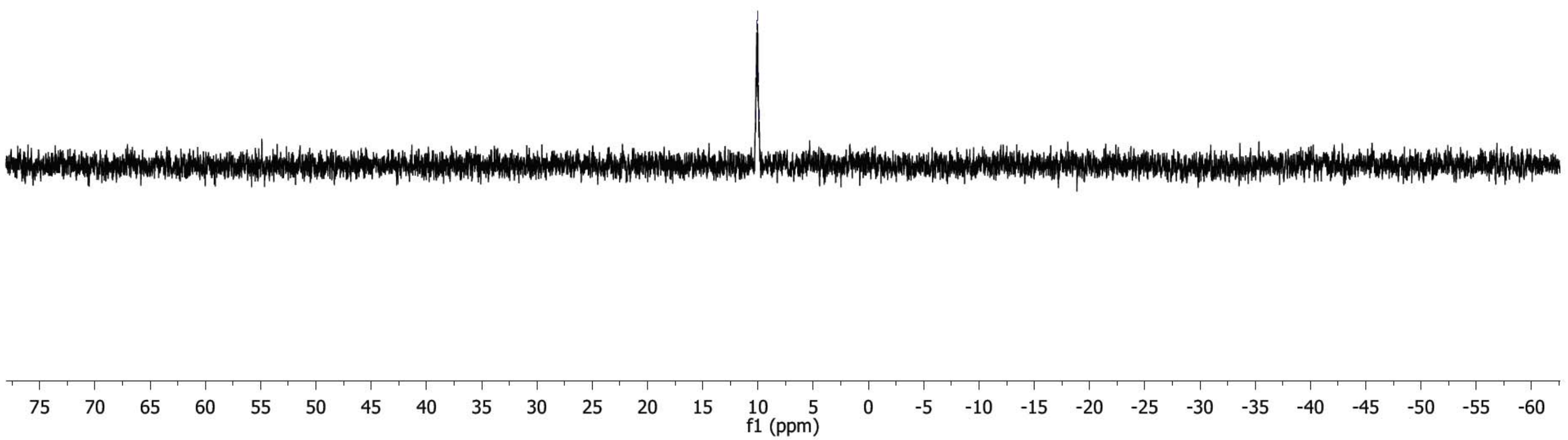

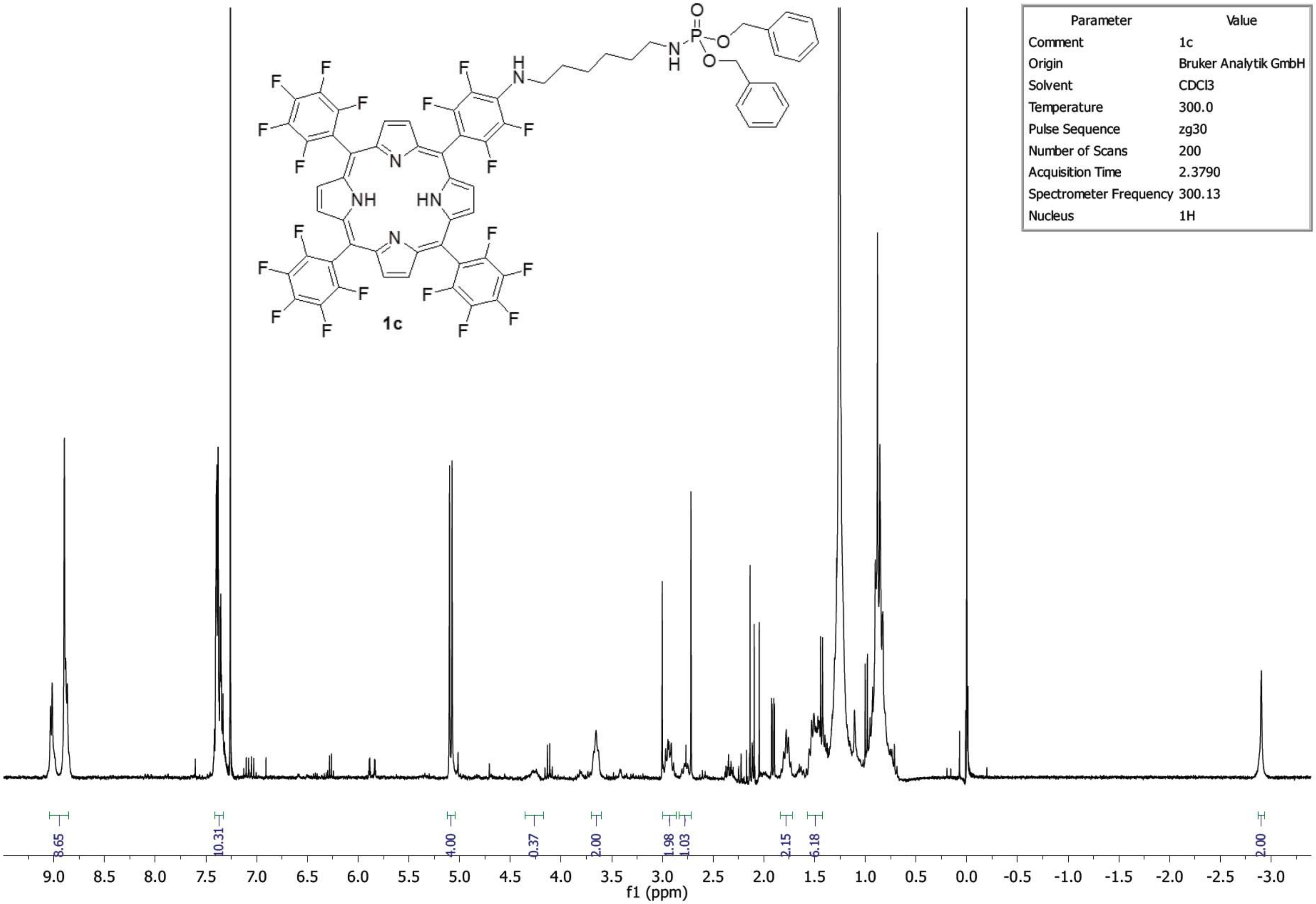
1c
Parameter Value
Comment 1c
Origin Bruker Analytik GmbH
Solvent CDCl3
Temperature 300.0
Pulse Sequence zg30
Number of Scans 200
Acquisition Time 2.3790
Spectrometer Frequency 300.13
Nucleus 1H
8.65
10.31
4.00
0.37
2.00
1.98
1.03
2.15
6.18
2.00
9.0 8.5 8.0 7.5 7.0 6.5 6.0 5.5 5.0 4.5 4.0 3.5 3.0 2.5 2.0 1.5 1.0 0.5 0.0 -0.5 -1.0 -1.5 -2.0 -2.5 -3.0
f1 (ppm)

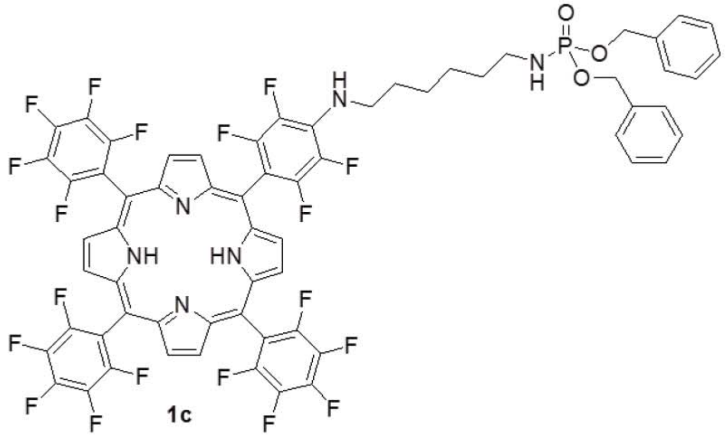

1c

Parameter	Value
Comment	1c
Origin	Bruker Analytik GmbH
Solvent	CDCl3
Temperature	300.0
Pulse Sequence	zg
Number of Scans	200
Acquisition Time	0.2901
Spectrometer Frequency	282.41
Nucleus	19F

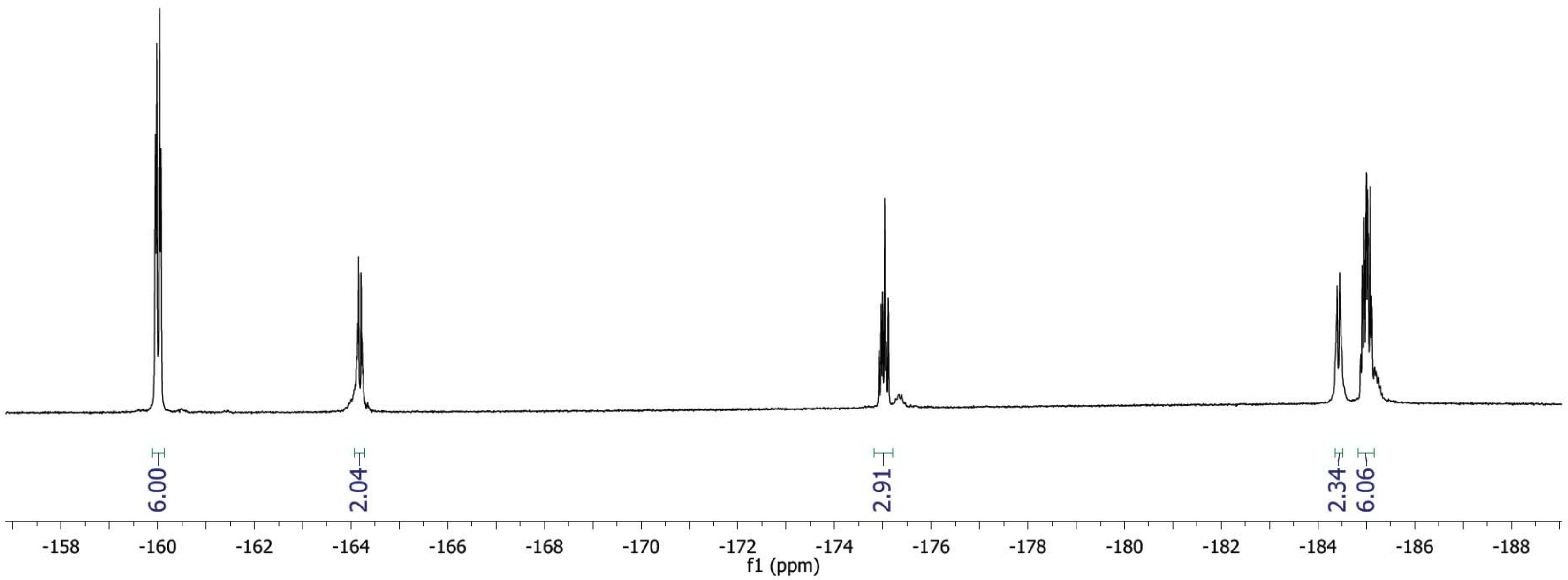

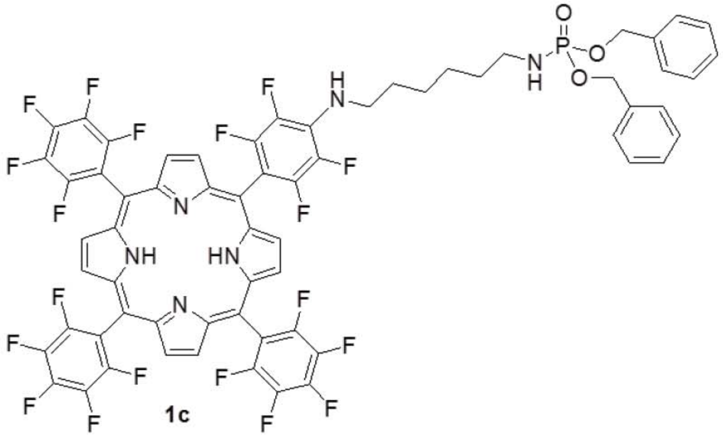

1c

Parameter	Value
Comment	1c
Origin	Bruker Analytik GmbH
Solvent	CDCl3
Temperature	300.0
Pulse Sequence	zg
Number of Scans	200
Acquisition Time	0.6751
Spectrometer Frequency	121.49
Nucleus	31P

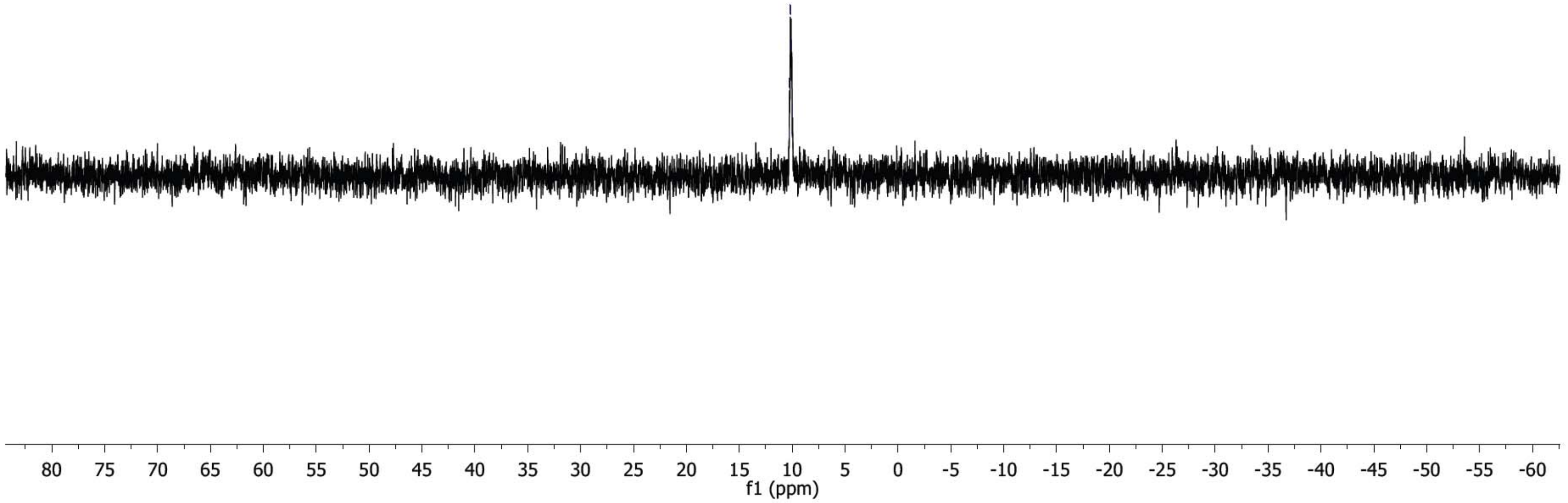

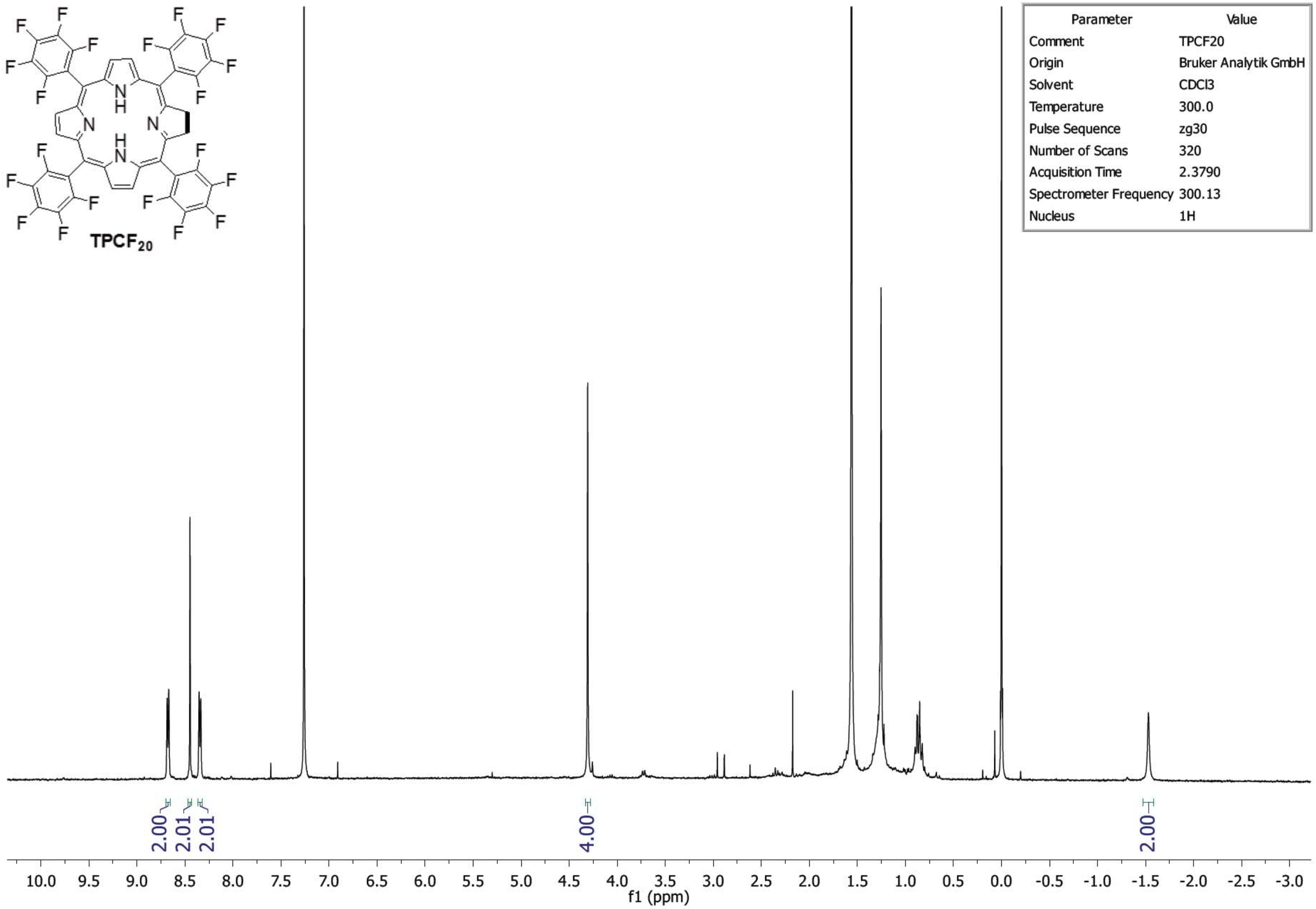
TPCF20
Parameter Value
Comment TPCF20
Origin Bruker Analytik GmbH
Solvent CDCl3
Temperature 300.0
Pulse Sequence zg30
Number of Scans 320
Acquisition Time 2.3790
Spectrometer Frequency 300.13
Nucleus 1H
2.00
2.01
2.01
4.00
2.00
10.0 9.5 9.0 8.5 8.0 7.5 7.0 6.5 6.0 5.5 5.0 4.5 4.0 3.5 3.0 2.5 2.0 1.5 1.0 0.5 0.0 -0.5 -1.0 -1.5 -2.0 -2.5 -3.0
f1 (ppm)

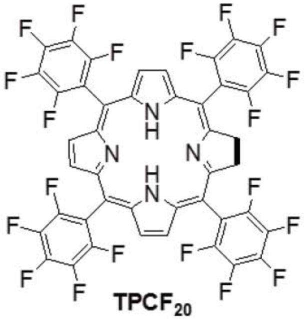

$TPCF_{20}$

Parameter	Value
Comment	TPCF20
Origin	Bruker Analytik GmbH
Solvent	CDCl3
Temperature	300.0
Pulse Sequence	zg
Number of Scans	320
Acquisition Time	0.2901
Spectrometer Frequency	282.41
Nucleus	19F

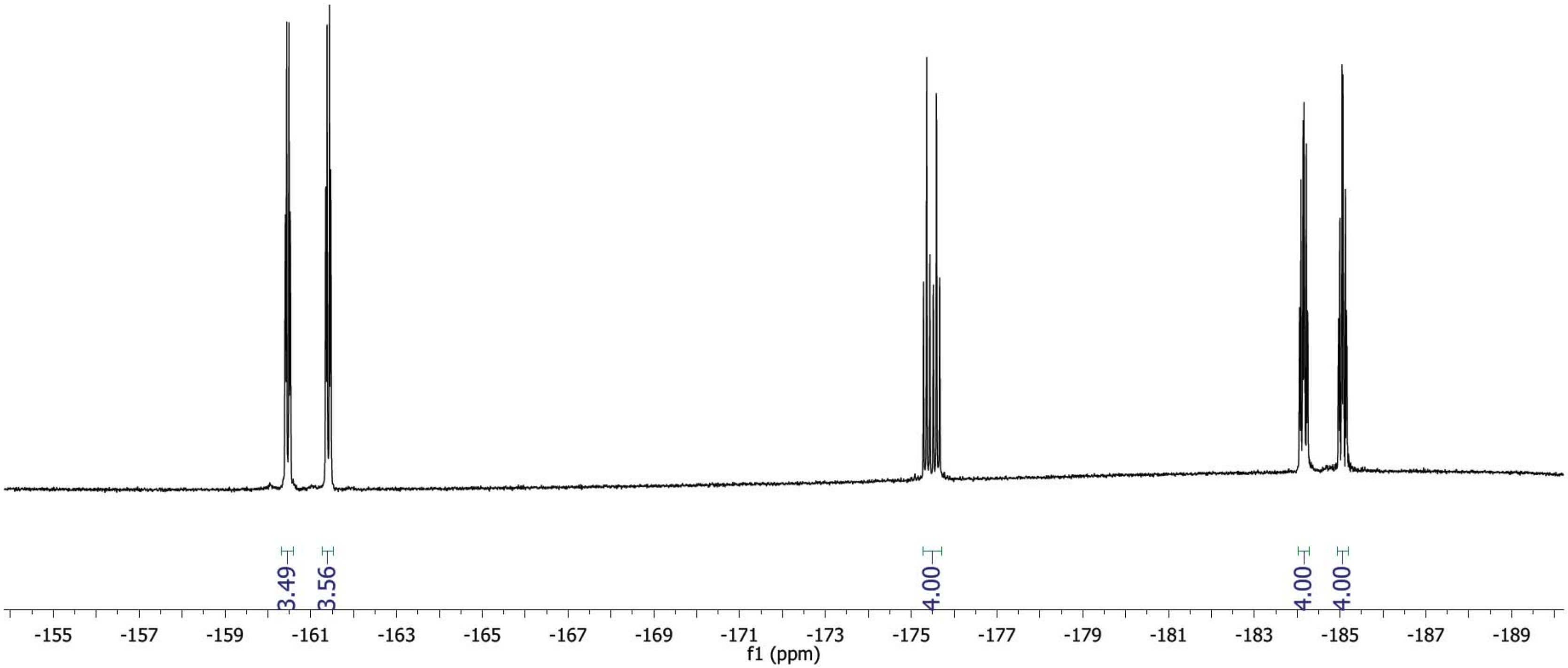

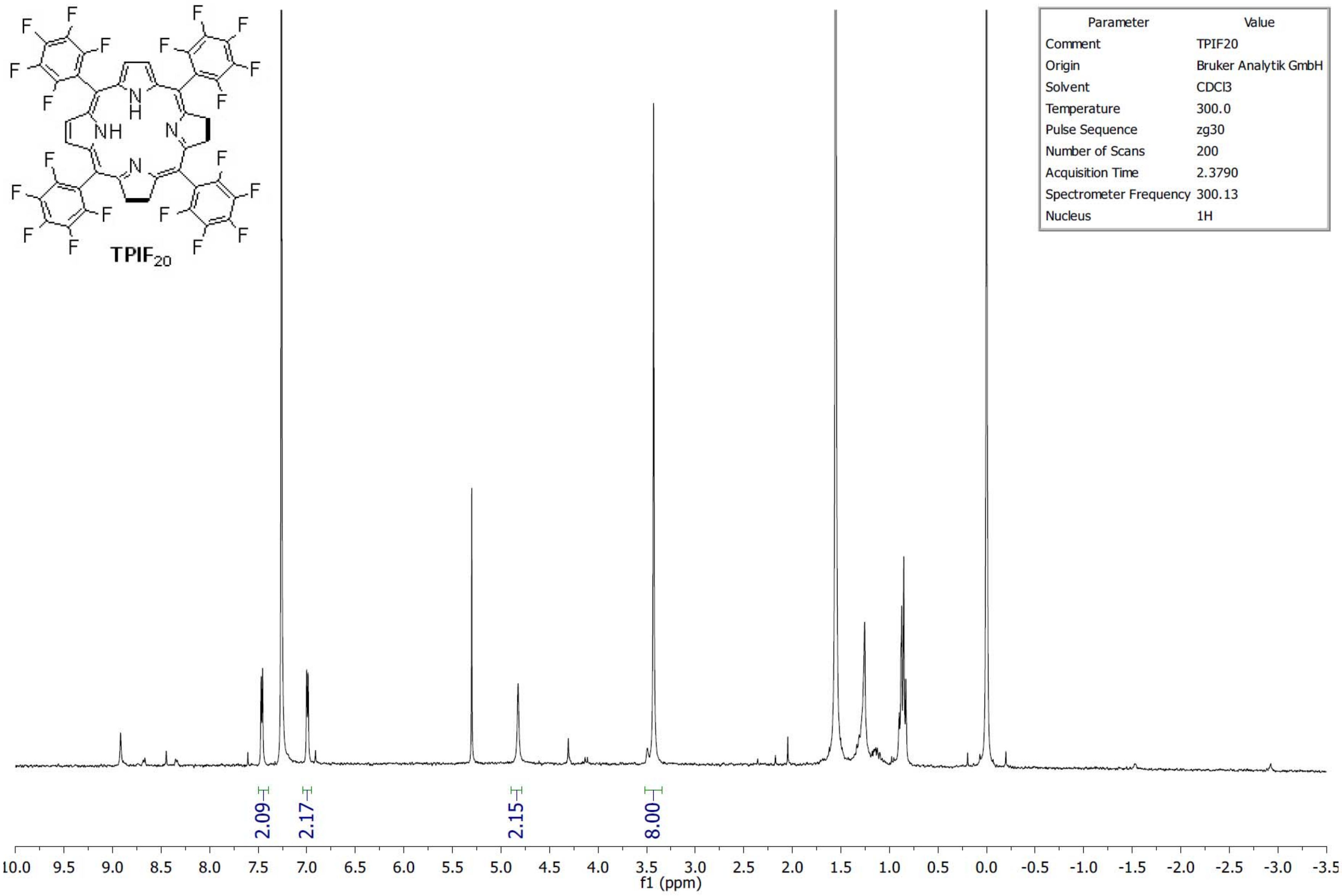

TPIF20
Parameter | Value
Comment | TPIF20
Origin | Bruker Analytik GmbH
Solvent | CDCl3
Temperature | 300.0
Pulse Sequence | zg30
Number of Scans | 200
Acquisition Time | 2.3790
Spectrometer Frequency | 300.13
Nucleus | 1H
2.09
2.17
2.15
8.00
10.0 9.5 9.0 8.5 8.0 7.5 7.0 6.5 6.0 5.5 5.0 4.5 4.0 3.5 3.0 2.5 2.0 1.5 1.0 0.5 0.0 -0.5 -1.0 -1.5 -2.0 -2.5 -3.0 -3.5
f1 (ppm)

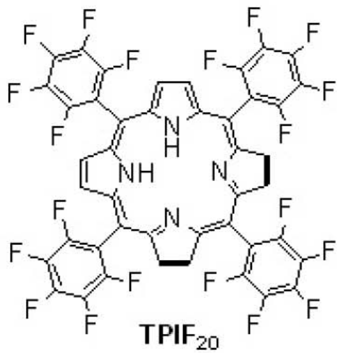

$TPIF_{20}$

Parameter	Value
Comment	TPIF20
Origin	Bruker Analytik GmbH
Solvent	CDCl3
Temperature	300.0
Pulse Sequence	zg
Number of Scans	200
Acquisition Time	0.2901
Spectrometer Frequency	282.41
Nucleus	19F

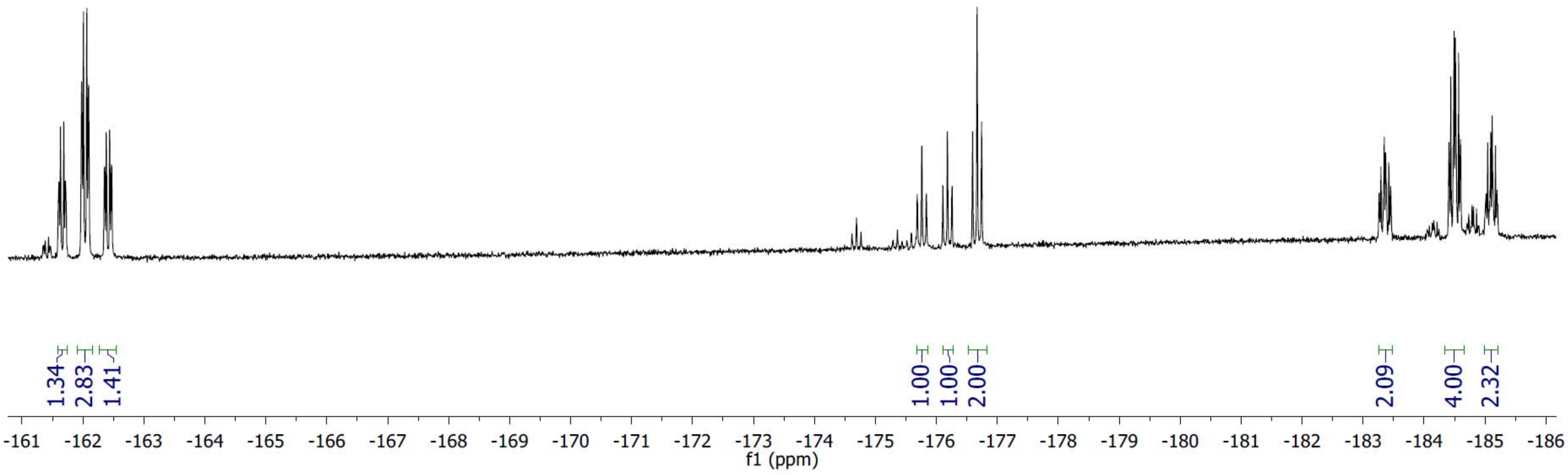

Supplement: File 1 — Experimental details, characterization data for new compounds, and copies of NMR spectra. [file Beilstein_J_Org_Chem-10-628-s001.pdf]
